# The Orthopaedic Trauma Patient Experience: A Qualitative Case Study of Orthopaedic Trauma Patients in Uganda

**DOI:** 10.1371/journal.pone.0110940

**Published:** 2014-10-31

**Authors:** Nathan N. O'Hara, Rodney Mugarura, Gerard P. Slobogean, Maryse Bouchard

**Affiliations:** 1 Department of Orthopaedics, University of British Columbia, Vancouver, British Columbia, Canada; 2 Department of Orthopaedics, Makerere University, Kampala, Uganda; 3 Department of Orthopaedics, Seattle Children's Hospital, Seattle, Washington, United States of America; Georgia Regents University, United States of America

## Abstract

The disability adjusted life years (DALYs) associated with injuries have increased by 34% from 1990 to 2010, making it the 10^th^ leading cause of disability worldwide, with most of the burden affecting low-income countries. Although disability from injuries is often preventable, limited access to essential surgical services contributes to these increasing DALY rates. Similar to many other low- and middle-income countries (LMIC), Uganda is plagued by a growing volume of traumatic injuries. The aim of this study is to explore the orthopaedic trauma patient's experience in accessing medical care in Uganda and what affects the injury might have on the socioeconomic status for the patient and their dependents. We also evaluate the factors that impact an individual's ability to access an appropriate treatment facility for their traumatic injury. Semi-structured interviews were conducted with patients 18 year of age or older admitted with a fractured tibia or femur at Mulago National Referral Hospital in Kampala, Uganda. As limited literature exists on the socioeconomic impacts of disability from trauma, we designed a descriptive qualitative case study, using thematic analysis, to extract unique information for which little has been previously been documented. This methodology is subject to less bias than other qualitative methods as it imposes fewer preconceptions. Data analysis of the patient interviews (n = 35) produced over one hundred codes, nine sub-themes and three overarching themes. The three overarching categories revealed by the data were: 1) the importance of social supports; 2) the impact of and on economic resources; and 3) navigating the healthcare system. Limited resources to fund the treatment of orthopaedic trauma patients in Uganda leads to reliance of patients on their friends, family, and hospital connections, and a tremendous economic burden that falls on the patient and their dependents.

## Introduction

The disability adjusted life years (DALYs) associated with injuries have increased by 34% from 1990 to 2010, making it the 10^th^ leading cause of disability worldwide, with most of the burden affecting low-income countries [1]. Although disability from injuries is often preventable, limited access to essential surgical services contributes to the increasing DALY rates [2], [3].

Similar to many other low- and middle-income countries (LMIC), Uganda is plagued by a growing volume of traumatic injuries. An estimated 50% of injuries in the country are due to road traffic collisions [4], [5]. Long bone fractures (femur and tibia) are the most common, non-fatal orthopaedic injuries accounting for over 15% of hospital admissions [6]. With timely and effective treatment of these injuries, a patient could expect a favourable recovery with minimal disability, as is seen and expected in high-income countries (HIC). However, scarce resources within the Ugandan and LMIC health systems leave many injured patients untreated or inadequately treated causing significant long-term disability.

Studies have highlighted the disproportionate prevalence and burden of orthopaedic trauma in males aged 18 to 45 [5], [7], [8]. What has less frequently been reported is that these males are often the sole breadwinners for a family of dependents. The health of this demographic is vital to the economic growth of a LMIC in both the short- and long-term [9]. In addition, this demographic is at higher risk of sustaining an injury. Increased labour participation, particularly in urban areas, put individuals at greater risk of road traffic accidents as they commonly commute on less safe means of transportation (small motorcycle taxis) [10].

Effective treatment of an injury is contingent upon timely admission to an appropriately resourced healthcare facility, and the capacity for that health facility to provide adequate treatment ultimately preventing disability. The pertinent provision of care enables people to fully recover and therefore stay economically productive, support their dependents and contribute to the economic growth of the country.

The findings presented represent one aspect of a study to qualify and quantify the socio-economic impact of orthopaedic trauma in Uganda. The aim of this branch of the study is to explore the orthopaedic trauma patient's experience in accessing medical care in Uganda and what affects the injury might have on the socioeconomic status of the patient and their dependents. We also evaluate the factors that impact an individual's ability to access an appropriate treatment facility for their traumatic injury.

## Methods

A descriptive qualitative case study was undertaken at Mulago National Referral Hospital in Kampala, Uganda. Data were collected through semi-structured interviews with patients 18 year of age or older admitted with a fractured tibia or femur. Patients with isolated lower extremity long bone fractures were selected, as it is well known that these injuries can cause significant disability; while mortality rates are lower than intracranial or poly-trauma [11]. Multi-trauma and head injury patients, were not only less likely to survive, but also less likely to be able to participate in the study. All patients who met the eligibility criteria during the study period were included in the sample. None of the patients who met the eligibility criteria refused to participant in the study.

Two authors conducted the interviews: NNO (male), a research manager with previous qualitative research experience and training, and RM (male), a clinical research fellow in orthopaedics with experience working and training in the Ugandan health system. Relationships between interviewers and patients were not established prior to the interviews. All interviews were semi-structured and conducted with the support of a local medical intern (female) who assisted as a translator. Each participant was only interviewed once and responses were recorded with a tablet survey tool (*QuickTap Survey, TabbleDaddle Inc., Toronto, Canada*) and augmented with interviewer notes. The patients were introduced to the interviewers and provided with a basic background on the study and the training of the researchers prior to the interviews. Written informed consent was obtained from all participants prior to the participation in the interview process. Interviews were conducted on the patient wards. Each interview lasted approximately 30 minutes. Family members of the patient were occasionally present during the interview. The interview questions were adapted from the 2009/10 National Panel Survey and augmented with clinical expertise from authorship group [12]. Data saturation was not discussed with the participants and interview transcripts or study findings were not presented to the patients for additional comments. Research ethics approval for this study was obtained from the University of British Columbia Clinical Research Ethics Board and the Mulago Research Ethics Committee. This study was conducted in compliance with the guidelines of the Helsinki Declaration.

As limited literature exists on the socioeconomic impact of disability from trauma, we designed a descriptive qualitative case study, using thematic analysis, to extract unique information for which little has been previously documented [13], [14], [15]. This methodology is subject to less bias than other qualitative methods as it imposes fewer preconceptions [16]. Data collected from the interviews was independently coded by two of the authors (NNO and MB). Initial inductive analysis discovered emergent themes and patterns in the data. Through authorship consensus, themes were condensed into overarching categories that represented and accurately explained the data set. Quotations used in the discussion were not identified to preserve confidentiality. Nvivo 9 Software was used to help facilitate management and analysis of the qualitative data.

## Results

Thirty-five interviews were conducted with patients who were admitted with a tibia or femur fracture to Mulago National Referral Hospital in Kampala, Uganda between September 30, 2013 and October 11, 2013. **Table 1** summarizes the characteristics of the study sample. Participants were predominantly male (86%) with a mean age of 42.7 years (SD = 17.5). Eighty-three percent of the participants were working at the time of injury but only 14% of the participants had a formal contract with their employer. 74% were the main breadwinner for their household supporting an average of 5.7 dependents. The median annual income for the study participants was $1200 (IQR: $276 - $4040). Most of the participants were injured by motor vehicles (80%), 69% arrived within 24 hours of their injury but only 23% of the admitted patients received treatment within two weeks of their injury. (A complete summary of all patient data is available in the **Table S1**.)

**Table 1:** Characteristics of study sample.

Characteristics		N	%
**Gender**			
	Male	**30**	86
**Age**			
	**Mean** = 42.7 years (**SD** = 17.5)		
**Occupation**			
	Business/Service	**13**	37
	Farmer	**7**	20
	Labourer	**6**	17
	Boda-Boda Driver*	**5**	14
	None	**4** **	11
**Annual Income**			
	Median = $1200 USD, IQR = ($276 - $4040)		
**Mechanism of Injury**			
	Multi-vehicle trauma	**13**	37
	Pedestrian–vehicle trauma	**10**	29
	Single-vehicle trauma	**5**	14
	Fall from standing	**4**	11
	Gun shot wound	**2**	6
	Violence (non-gun)	**1**	3
**Time from Injury to Admission**			
	Less than 24 hrs	**24**	69
	24 – 48 hrs	**2**	6
	48-72 hrs	**1**	3
	3 – 10 days	**5**	14
	More than 10 days	**3**	9

(n = 35).

*Boda-Bodas are motorcycles used to taxi people.

** 3 of 4 of these participants were aged ≥64 years.

**Figure 1:**
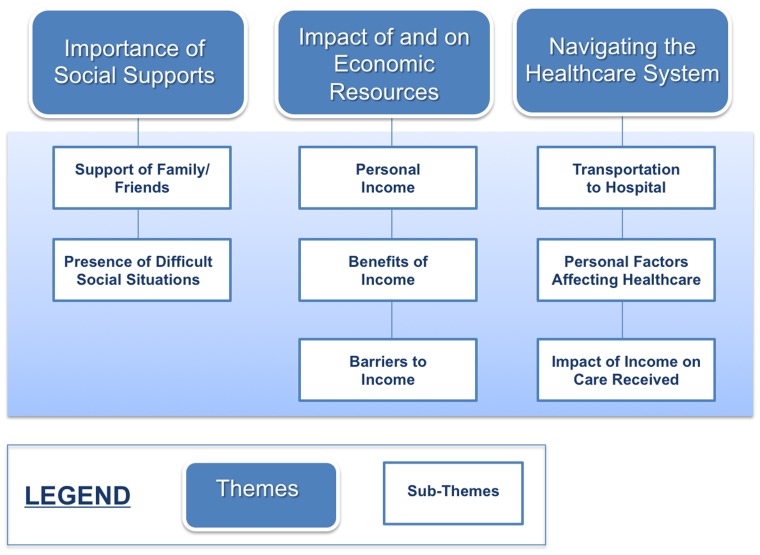
Themes and sub-themes derived from qualitative analysis.

Data analysis produced over one hundred codes, nine sub-themes and three overarching themes. A matrix of dominant and important themes can be found in **Table 2**. The three overarching categories revealed by the data were: 1) the importance of social supports; 2) the impact of and on economic resources; and 3) navigating the healthcare system (**Figure 1**). Sub-themes discovered within the importance of social supports were support of family and friends and the presence of difficult social situations. The impact of and on economic resources themes were distilled into three sub-themes: personal income, benefits of income and barriers to income. Transportation to hospital, personal factors affecting healthcare and impact of income on care received were the sub-themes derived from navigating the healthcare system, the third overarching theme. (A complete summary of all codes and their frequencies is available in the **Table S2**.).

**Table 2:** Sample of codes and themes from interview data.

Line-by-line Code	Frequency of code
Supports children	21
Supports spouse	20
Direct admission to Mulago Hospital	18
Transfer to Mulago Hospital from another hospital	14
Children in school	12
No treatment at “other” hospital	12
Immediate transfer to hospital from injury site	11
Supports siblings	10
Waiting on ward for treatment/Delayed treatment	8
Makes decent/good money	7
Diploma/degree	7
Little money/income	5

## Discussion

In Uganda, like many other LMICs, an individual's socioeconomic status has a profound impact on their ability to access the healthcare system and to receive timely treatment for an injury [10]. There are many potential barriers to hospital admission and treatment for injured patients in Uganda. Our findings identified common barriers to hospital admission such as lack of available funds for transportation and the patient's proximity to an appropriately equipped health facility. Interestingly, these impediments are often different from the patient's expressed barriers to treatment, which include limited social support and a lack of social contacts working within health system.

### Social Supports

Qualitative analysis highlighted two dominant themes associated with the social supports of injured patients: 1) the support of friends and family and 2) the presence of difficult social situations. Traumatic injuries were found to not only have a substantial impact on the patient but also on their family and friends. 83% of the patients in the study were working at the time of injury, often supporting a spouse, children and siblings. Following the injury, which rendered most patients unable to work, many patients were unable to pay the school fees of their children. Furthermore, surgical wards have limited nursing care. Injured patients had to rely on those dependents for hygiene and feeding. One elderly patient noted his son would have to support all of his medical costs. The presence of difficult social situations emerged during the analysis as a second sub-theme. Patients spoke of the struggle associated with the death of a family member in the same traumatic event, the anxiety associated with struggling to support dependents during the time of injury, and burden of unrelated illness and mental health conditions.

### Navigating Access to Care

Data analysis revealed important sub-themes relevant to navigating the Ugandan health system after a traumatic injury. A greater understanding of the relationship between minimal income and delays to hospital admission were discovered. One patient delayed travelling to the hospital for almost a month after a femur fracture due to concern about the cost of transport to the hospital in Kampala and the presumed cost of treatment. Some patients noted they had limited information on availability of appropriate health services. One patient waited two weeks for an orthopaedic consultant to visit a regional hospital and eventually voluntarily arranged to be transported to Kampala for a consultation. Other patients noted they would seek care based on the location of their family support network. A patient from Arua, a town in the northwest of the country, was given the option to be admitted in nearby Gulu (a 3-hour drive) or Kampala (a 8-hour drive). Even though Kampala was a farther, more expensive trip, the individual chose to travel to Kampala because they had family there.

### Economic Burden of Catastrophic Injury

Of the 83% of study participants that were working at the time of injury, 74% were the main breadwinner for their household supporting an average of 5.7 dependents. One patient, a peasant farmer, supports his family by selling excess yield at the local market. His last sale of five months of effort provided the equivalent of $12 USD. Another patient was informally employed but unable to earn enough money to support his wife and three children, so he attempted to steal fuel to sell for extra income but was shot in the leg by an on-site security officer.

Economic activity in Uganda is largely derived from the informal sector. The informal sector of the economy refers all economic activities by workers that are not covered or insufficiently covered by formal arrangements [17]. Those employed in the informal sector are typically not privileged to forms of social insurance and pension. Wages are typically provided based on work completed and can be suspended frequently and without notice. A catastrophic injury to an individual who is informally employed, not only has a profound impact on the injured individual, but also greatly affects the dependents of that individual. Informal economies are pervasive in many low-income countries with potentially negative consequences. These consequences may include decreased competitiveness and growth, incomplete coverage of formal social programs, undermining social cohesion and law and order, and fiscal losses due to undeclared economic activity impeding the overall economic growth and impacting economic equality [18]. Participants in the informal economy do not have their income taxed directly. Therefore, increased economic productivity in this segment of the economy does not support the growth of public services such as government-funded health care. A strong formal economy fuels a social safety net that supports citizens through catastrophic events.

It is worth noting that even if a patient is admitted to a hospital shortly after injury, limited funding for the health system often requires them to buy the implant needed for their treatment. The costs of standard trauma implants for managing long bone fractures are high: a 10-hole plate and screw construct is $300 USD and a femoral intramedullary rod is $900 USD [19]. One patient commented that he has little chance of being able to buy the necessary implant for his treatment and will most likely have to be treated with traction, a sub-optimal method which renders a patient bed-ridden for months while the fracture heals.

Those patients with dependents routinely commented that their hospitalization and subsequent withdrawal from the workforce during recovery had a devastating affect on their dependents. Injured breadwinners were faced with difficult decisions on what treatment options they could afford balanced with the financial needs of their dependents. The patient often lacked the adequate savings to continuing paying for their children's school fees during their hospital stay, forcing them to withdraw from school at least temporarily.

### Study Limitations

The study particants were interviewed within two weeks of admission. A greater understanding of socioeconomic issues related to a traumatic injury may be identified with a longer period of data collection. It is reasonable to assume that the longer a person waits on the wards without treatment, the greater their motivation to seek available economic resources to facilitate a faster treatment where they are admitted or transfer to a private hospital for more immediate treatment.

We acknowledge that the study is limited to those patients who were eventually admitted to the country's tertiary hospital. There are most likely many other patients that did not make it to the appropriate health facility or declined to seek treatment due to an inability to bear the costs of care. A complete analysis of the socio-economic effects on trauma patients would require the ability to interview all injured people, including those who have not been able to access appropriate medical care. Considering the inadequacy of emergency medical services in Uganda and the large number of smaller medical facilities and clinics, identifying all patients would be difficult. We therefore consider our smaller, focused study sample to be a good primary index and investigation into the barriers to care and impacts of injury.

### Suggestions for Further Research

This field of research would benefit from further insight into the long-term socio-economic implications of traumatic injuries. Evaluating if these patients return to the same level of economic productivity, if they maintain the same socio-economic status post-injury and the impact of these injuries on their dependents would be valuable information for policy makers. There are many potential factors that will contribute to a patient's recovery. For instance, one patient noted that his brothers have good jobs and will be able to support him while he recovers. This seemed like an unfortunately rare circumstance among this patient population.

An increased awareness of the factors that lead to the timely and effective treatment of orthopaedic injuries in low-income countries will enable government and policy makers to create effective interventions for improving trauma care. We have found that a strong social support system for the patient, the ability of the patient to understand and navigate the health system, and the impact of an injured breadwinner on that individual and their dependents, are crucial elements in determining whether an injured patient will receive timely, appropriate care.

### Conclusion

The appreciation of the association between traumatic injuries and their implications for economic growth should lead to improved resource allocation for both prevention and treatment these injuries. Improved mechanisms to fund the management of treating trauma patients (government funding, availability of loans, access to insurance) would potentially alleviate the present reliance of patients on their friends, family, and hospital connections, and diminish the economic burden that falls on the patient and their dependents.

## Supporting Information

Table S1 Data Set Characteristics.
(PDF)

Table S2 Code Frequencies.
(PDF)

Table S3 Qualitative Interview Questions.
(DOCX)

Table S4 COREQ Checklist.
(DOCX)

## References

[1] Murray CJ, Vos T, Lozano R, Naghavi M, Flaxman AD, et al (2012) Disability-adjusted life years (DALYs) for 291 diseases and injuries in 21 regions, 1990–2010: a systematic analysis for the Global Burden of Disease Study 2010. Lancet 380(9859): 2197–2223. 23245608 10.1016/S0140-6736(12)61689-4

[2] Weiser TG, Regenbogen SE, Thompson KD, Haynes AB, Lipsitz SR, et al (2008) An estimation of the global volume of surgery: a modelling strategy based on available data. Lancet 372(9633): 139–144. 18582931 10.1016/S0140-6736(08)60878-8

[3] Grimes CE, Bowman KG, Dodgion CM, Lavy CBD (2011) Systematic review of barriers to surgical care in low-income and middle-income countries. World J Surg 35(5): 941–950. 21360305 10.1007/s00268-011-1010-1

[4] Demyttenaere SV, Nansamba C, Nganwa A, Mutto M, Lett R, et al (2009) Injury in Kampala, Uganda: 6 years later. Can J Surg 52(5): E146–150. 19865544 PMC2769114

[5] Hsia RY, Ozgediz D, Mutto M, Jayaraman S, Kyamanywa P, et al (2010) Epidemiology of injuries presenting to the national hospital in Kampala, Uganda: implications for research and policy. Int J Emerg Med 3(3): 165–172. 21031040 10.1007/s12245-010-0200-1 PMC2926872

[6] Mock C, Cherian MN (2008) The global burden of musculoskeletal injuries: challenges and solutions. Clin Orthop Relat Res 466(10): 2306–2316. 18679760 10.1007/s11999-008-0416-z PMC2584305

[7] Kobusingye OC, Guwatudde D, Owor G, Lett RR (2002) Citywide trauma experience in Kampala, Uganda: a call for intervention. Inj Prev 8(2): 133–136. 12120832 10.1136/ip.8.2.133 PMC1730841

[8] Galukande M, Jombwe J, Fualal J, Gakwaya A (2009) Boda-boda injuries a health problem and a Burden of Disease in Uganda: A tertiary Hospital survey. East Central Afr J Surg 14(2): 33–37.

[9] Bloom DE, Canning D, Sevilla J (2004) The effect of health on economic growth: a production function approach. World Dev 32(1): 1–13.

[10] Nantulya VM, Reich MR (2002) The neglected epidemic: road traffic injuries in developing countries. Br Med J 324(7346): 1139. 12003888 10.1136/bmj.324.7346.1139 PMC1123095

[11] Begg S, Tomijima N, Vos T, Mathers CD (2003) Global burden of injury in the year 2000: an overview of methods. Geneva: WHO.

[12] The World Bank (2011) Uganda 2011/12 national panel survey. Available: http://econ.worldbank.org/WBSITE/EXTERNAL/EXTDEC/EXTRESEARCH/EXTLSMS/0, contentMDK:23511127∼menuPK:4196952∼pagePK:64168445∼piPK:64168309∼theSitePK:3358997∼isCURL:Y,00.html. Accessed 10 June 2014.

[13] Keen J, Packwood T (1995) Case study evaluation. Br Med J 311(7002): 444. 7640596 10.1136/bmj.311.7002.444 PMC2550500

[14] Fereday J, Muir-Cochrane E (2006) Demonstrating rigor using thematic analysis: A hybrid approach of inductive and deductive coding and theme development. Int J Qual Methods 5(1): 80–92.

[15] Braun V, Clarke V (2006) Using thematic analysis in psychology. Qual Res Psychol 3(2): 77–101.

[16] Sandelowski M (1991) Telling stories: Narrative approaches in qualitative research. J Nurs Scholarsh 23(3): 161–166. 10.1111/j.1547-5069.1991.tb00662.x 1916857

[17] International Labour Organization (2012) Resource guide on the informal economy. Available: http://www.ilo.int/public/english/support/lib/resource/subject/informal.htm. Accessed 10 May 2014.

[18] The World Bank (2013) Workers in the informal economy. Available: http://web.worldbank.org/WBSITE/EXTERNAL/TOPICS/EXTSOCIALPROTECTION/EXTLM/0, contentMDK:20224904∼menuPK:584866∼pagePK:148956∼piPK:216618∼theSitePK:390615,00.html. Accessed 25 May 2014.

[19] Bouchard M (2011) Responding to the global injury burden by improving access to orthopaedic medical devices: A qualitative case study of orthopaedic services in Uganda. M.Sc. Thesis, The University of Toronto. Available: https://tspace.library.utoronto.ca/bitstream/1807/30520/6/Bouchard_Maryse_201111_MSc_Thesis.pdf. Accessed 10 June 2014.

