# Supplementary material for: The Orthopaedic Trauma Patient Experience: A Qualitative Case Study of Orthopaedic Trauma Patients in Uganda
Source: PLoS One. 2014 Oct 31;9(10):e110940. doi: 10.1371/journal.pone.0110940 (PMC4215992; doi:10.1371/journal.pone.0110940)
Supplement: Table S1 — Data Set Characteristics. (PDF) [file pone.0110940.s001.pdf]

Complete summary of all patient data

**Demographics**			
	**Sex**, n (%)		
		Male	30 (85.7)
		Female	5 (14.2)
	**Age**, n (%)		
		18-19	1 (2.9)
		20-29	8 (22.9)
		30-39	8 (22.9)
		40-49	7 (20.0)
		50-59	4 (11.4)
		60-69	3 (8.6)
		70-79	3 (8.6)
		80-89	1 (2.9)
**Household Data**			
	**Dependents**		
		Mean (SD)	5.7 (3.5)
	**Type of home**, n (%)		
		Independent house	16 (45.7)
		Boys quarters	8 (22.9)
		Tenement (Muzigo)	5 (14.3)
		Hut	3 (8.6)
		Sharing house/flat/apartment	2 (5.7)
		Other	1 (2.9)
	**Stairs in home**, n (%)		
		Yes	4 (11.4)
		No	31 (88.6)
	**Electricity in home**, n (%)		
		Yes	20 (57.1)
		No	15 (42.9)
	**Source of drinking water for home,** n (%)		
		Public taps	11 (31.4)
		Private connection	8 (22.9)
		Bore-hole	7 (20.0)
		Protected well/spring	5 (14.2)
		Unprotected well/spring	3 (8.6)
		River, stream, lake, pond	1 (2.9)
	**Marital status**, n (%)		
		Married	25 (71.4)
		Never married	7 (20.0)
		Undisclosed	2 (5.7)
		Divorced	1 (2.9)

	**Education level**, n (%)		
		No education	1 (2.9)
		Primary education	19 (54.2)
		Secondary education	11 (31.4)
		Diploma/technical school	2 (5.7)
		Degree	2 (5.7)
	**Years in current home**		
		Median (IQR)	9 (5-20)
	**Distance from home to Mulago Hospital** (km)		
		Median (IQR)	11 (6 – 80)
**Economic Data**			
	**Main income earner**, n (%)		
		Patient	26 (74.3)
		Child of patient	4 (11.4)
		Parent of patient	3 (8.6)
		Other	2 (5.7)
	**Employment situation prior to injury,** n (%)		
		Working	29 (82.9)
		Taking care of house/family	4 (11.4)
		In school	1 (2.9)
		Disabled	1 (2.9)
	**Occupation**, n (%)		
		Business/Service	13 (37.1)
		Farmer	7 (20.0)
		Labourer	6 (17.1)
		Boda-boda driver	5 (14.3)
		Not employed	4 (11.4)
	**Formal contract**, n (%)		
		No	30 (85.7)
		Yes	5 (14.3)
	**Annual Income**, USD		
		Median (IQR)	$1200 ($276 - $4040)
	**Other sources of income**, n (%)		
		Remittance	1 (2.9)
		Church	1 (2.9)
		None	33 (94.3)
**Injury Data**			
	**Mechanism of injury**, n (%)		
		Multi-vehicle trauma	13 (37.1)
		Pedestrian-vehicle trauma	10 (28.6)
		Single-vehicle trauma	5 (14.3)
		Fall from standing	4 (11.4)

		Gun shot wound	2 (5.7)
		Violence (non-gun)	1 (2.9)
	**Time: Injury to Mulago Hospital**, n (%)		
		Less than 24 hrs	24 (68.6)
		24 – 48 hrs	2 (5.7)
		48 – 72 hrs	1 (2.9)
		3 – 10 days	5 (14.3)
		More than 10 days	3 (8.6)
	**Treatment within two weeks post injury**, n (%)		
		Yes	8 (22.8)
		No	27 (77.1)
	**Patient admitted from**:, n (%)		
		Injury site	14 (40.0)
		Other hospital	13 (37.1)
		Home	5 (14.3)
		Local clinic	3 (8.6)
	**Method of transport to Mulago Hospital**, n (%)		
		Motor vehicle	24 (68.6)
		Ambulance	8 (22.9)
		Boda-boda	2 (5.7)
		Other	1 (2.9)
