# Supplementary material for: The Orthopaedic Trauma Patient Experience: A Qualitative Case Study of Orthopaedic Trauma Patients in Uganda
Source: PLoS One. 2014 Oct 31;9(10):e110940. doi: 10.1371/journal.pone.0110940 (PMC4215992; doi:10.1371/journal.pone.0110940)
Supplement: Table S2 — Code Frequencies. (PDF) [file pone.0110940.s002.pdf]

	Line-by-line Code	Frequency of code	Number of references
1	Supports/lives with children	21	21
2	Supports/lives with spouse	20	20
3	Femur fracture	20	20
4	Direct to Mulago (trauma) Hospital	18	18
5	Boda boda accident	14	14
6	Transfer to Mulago from other hospital	14	14
7	Children in school	12	12
8	No treatment at “other” hospital	12	12
9	Tibia fracture	11	11
10	Immediate transfer to hospital from injury site	11	11
11	Supports/lives with siblings	10	10
12	Hit by vehicle/bicycle	10	10
13	Open fracture	8	8
14	Waiting on ward for treatment/ Delayed treatment	8	8
15	Makes decent/good money	7	7
16	Diploma/degree	7	7
17	Farmer	6	6
18	Main income earner	5	5
19	Little money/income	5	5
20	Taxi/boda boda driver	4	4
21	Sell products at market	4	4
22	Difficulty finding employment/irregular income	4	3
23	Supports/lives with grandchildren	4	4
24	Owns (food vending) shop	4	4
25	Access to money	3	3
26	Hemi-arthroplasty	3	3
27	Private room	3	3
28	Well educated *(included in degree code also)*	3	3
29	External fixator	3	3
30	Financial support from parents	3	3
31	Unemployed	3	3
32	Helps on farm/around house	3	3

33	Wait at home before going to hospital because of concerns of costs	3	3
34	Bus accident	3	3
35	Fall	3	3
36	Mechanic	2	2
37	Security officer	2	2
38	Widow	2	2
39	Employer to pay medical bills	2	2
40	Plate fixation	2	2
41	Supports others' children	2	2
42	Grandparent	2	2
43	Up-country	2	2
44	No employees	2	2
45	Supports/lives with parent	2	2
46	Financial support by children	2	2
47	Death of family member	2	2
48	Robbery	2	2
49	Gunshot	2	2
50	Tuberculosis	2	2
51	Technician	2	2
52	Traction/Skin traction	2	2
53	Injury while working	2	2
54	Can't afford school for children	2	2
55	Financial support from family	2	2
56	Family support during rehabilitation/hospitalization	2	2
57	Husband-and-wife owned business	2	2
58	Microbiologist	1	1
59	Contacts at hospital	1	1
60	Wasn't working	1	1
61	Ambulance	1	1
62	No treatment up-country	1	1
63	No income while in hospital	1	1
64	RTA	1	1
65	Laborer	1	1
66	Fisheries officer	1	1
67	Delayed pain management	1	1
68	Private hospital	1	1
69	Kids out of school while	1	1

	parent in hospital		
70	Porter	1	1
71	Chronic lung disease	1	1
72	IM nail	1	1
73	Cancer	1	1
74	Barber	1	1
75	Police officer	1	1
76	Driving too fast	1	1
77	Braids hair for income	1	1
78	Financial support from late spouse	1	1
79	Loans/scholarships for school	1	1
80	Financial support from friend	1	1
81	Hope injury will not require medical attention	1	1
82	Divorced	1	1
83	Travel extra distance to be near family during hospitalization	1	1
84	Tailor	1	1
85	Much younger spouse	1	1
86	Live off one's farm	1	1
87	Welder	1	1
88	Starts going to church after injury to avoid future bad luck such as the accident	1	1
89	Alcohol abuse	1	1
90	Marijuana use	1	1
91	Intoxicated at time of injury	1	1
92	Schizophrenia	1	1
93	Co-incident illness making it hard to work	1	1
94	Lives alone	1	1
95	Not married	1	1
96	Has employees	1	1
97	Attacked/in a fight	1	1
98	Construction worker	1	1
99	Transfer between hospitals by personal car	1	1
100	From South Sudan	1	1
101	Own a bar	1	1
102	Move to Uganda for improved schooling for kids	1	1

103	DJ	1	1
104	Employee of large company	1	1
