# Supplementary material for: The Orthopaedic Trauma Patient Experience: A Qualitative Case Study of Orthopaedic Trauma Patients in Uganda
Source: PLoS One. 2014 Oct 31;9(10):e110940. doi: 10.1371/journal.pone.0110940 (PMC4215992; doi:10.1371/journal.pone.0110940)
Supplement: Table S3 — Qualitative Interview Questions. (DOCX) [file pone.0110940.s003.docx]

**Interview Questions**

**Staff Details**

1. Name of Interviewer:
2. Date of Interview:
3. Start Time of Interview:
4. Response Code: 1^st^ Visit
5. Completed
6. Partially Done
7. Not Complete
8. Reason for non-participation:

**Patient Details**

1. Study Number
2. Gender
3. Date of Birth
4. Country of Residence
5. Home City/Village

**Injury Details**

1. Type of Injury
2. Mechanism of Injury
3. Time of Injury
4. Date of Injury
5. Hospital/Clinic of First Assessment
6. Additional Centers of Treatment
7. Transportation Method to Mulago Hospital
8. Did anyone accompany them to the hospital?
   1. The relationship of that person?
   2. Will they continue to remain with them at the hospital?
9. Date of Arrival at Mulago Hospital
10. Time of Arrival at Mulago Hospital

**Household Information**

1. What is the roster of household members in the last 12 months.
2. What is the relationship of members to the head of the household.
3. What are the ages of household members?
4. During the past 12 months, how many months did the patient live in this home?
5. What is the residential status of the patient?
   1. Usual member
   2. Usual member, absent
   3. Guest
   4. Usual member who has left household more than six months
   5. Left permanently
6. What type of dwelling does the patient live in:
   1. Independent house
   2. Tenement (Muzigo)
   3. Independent flat/apartment
   4. Sharing house/flat/apartment
   5. Boys quarters
   6. Garage
   7. Hut
   8. Uniport
   9. Other (specify)
7. What is the present marital status of the patient?
8. What is the highest level of education completed by each member of the household?

**Economic Information**

1. Who is the main income earner for the household?
2. Were any other household members injured in the same incident?
   1. Do they contribute to the household income?
3. In the last week, did the patient work for a wage, salary, commission or any payment in kind, even if done for only one hour?
4. Did the patient do this type of work in the last 12 months?
5. In the last week, did the patient run a business of any size, for themselves or another household member, even if for only one hour?
6. Did the patient run a business in the last 12 months?
7. In the last week, did the patient help without being paid in any kind of business run by this household, even if for only one hour?
8. Did the patient do this in the last 12 months?
9. In the last week, did the patient work on their household farm?
10. Did the patient work on the household’s farm in the 12 months?
11. If the patient did not work/apprentice/support a household business in the past 7 days, does the patient have a job or business they will be returning to?
12. In the last four weeks, was the patient looking for any kind of job?
13. In the last four weeks, was the patient trying to start any kind of business?
14. What best describes the patient’s situation last week?
    1. Ill/sick
    2. Disabled
    3. In school
    4. Taking care of a house or family
    5. Retired
    6. Waiting for reply from employer
    7. Waiting for busy season
    8. Working
    9. Other (specify)

**MAIN JOB**

1. What kind of work does the patient usually do in their main job/business that the patient had during the last week?
2. What are the main goods/services produced at the patient’s place of work or its main function?
3. When did the patient start to work for this employer or start running the business?
4. In this main job/business that the patient had during the last week, was:
   1. Working for some else for pay?
   2. An employer?
   3. An own-account worker?
   4. Helping without pay in a household business?
   5. An apprentice?
   6. Working on the household farm or with household livestock?
5. Does this employer contribute to any pension/retirement fund (eg. NSSF) for the patient?
6. Is the patient entitled to any paid leave from employer?
7. Is the patient entitled to medical benefits from this employer?
8. Does the employer deduct or pay income tax from the patient’s wage/salary?
9. Is the patient’s employment agreement:
   1. Written
   2. Verbal
10. What is the duration of the patient’s employment agreement?
    1. A week or less
    2. More than a week but less than a month
    3. One to six months
    4. Seven to eleven months
    5. One to five years
    6. More than five years
11. Is the patient’s position:
    1. Permanent and pensionable
    2. An open ended appointment
    3. A fixed term
    4. None of the above
12. During the last 12 months, for how many months did the patient work in this job?
13. How much was the patient’s last cash payment and estimated value of what the patient last received in kind of the main job during the last week?

Cash:

Estimated cash value of in-kind payments:

1. What time period did the payment cover?
   1. Hour
   2. Day
   3. Week
   4. Month
   5. Other (specify)
2. Is the patient’s employer/business:
   1. National government
   2. Local government
   3. Government controlled business
   4. A commercial bank
   5. A private enterprise (other than a commercial bank)
   6. Non-profit organization
   7. A private household
3. During the last seven days, how many hours did the patient work in each day?

Sun

Mon

Tues

Wed

Thurs

Fri

Sat

1. In the last week, did the patient have more than one economic activity, such as a job, business, household enterprise or farm?
2. Does the patient get income or support from any of the following sources?
   1. Remittances
   2. Charity/church
   3. Retirement pension
   4. NSSF
   5. Welfare grants
   6. Bursary/student loan
   7. Other (specify)
   8. None
3. What is the value of that support in the last 12 months?
