# Supplementary material for: The Orthopaedic Trauma Patient Experience: A Qualitative Case Study of Orthopaedic Trauma Patients in Uganda
Source: PLoS One. 2014 Oct 31;9(10):e110940. doi: 10.1371/journal.pone.0110940 (PMC4215992; doi:10.1371/journal.pone.0110940)
Supplement: Table S4 — COREQ Checklist. (DOCX) [file pone.0110940.s004.docx]

**COREQ Checklist**

The orthopaedic trauma patient experience: A qualitative case study of orthopaedic trauma patients in Uganda

| **No** | **Item** | **Guide questions/description** |  |
| --- | --- | --- | --- |
| **Domain 1: Research team and reflexivity** | | |  |
| Personal Characteristics | | |  |
| 1. | Interviewer/facilitator | Which author/s conducted the interview or focus group? | NNO and RM |
| 2. | Credentials | What were the researcher's credentials? *E.g. PhD, MD* | NNO – MHA  RM - MMed |
| 3. | Occupation | What was their occupation at the time of the study? | NNO – Research Manager  RM – Clinical Research Fellow, Orthopaedics |
| 4. | Gender | Was the researcher male or female? | NNO and RM are both male, translation support was female |
| 5. | Experience and training | What experience or training did the researcher have? | NNO – has previous qualitative research experience and training in qualitative research  RM – research training and familiarity with health context |
| Relationship with participants | | |  |
| 6. | Relationship established | Was a relationship established prior to study commencement? | No |
| 7. | Participant knowledge of the interviewer | What did the participants know about the researcher? e*.g. personal goals, reasons for doing the research* | The patients were introduced to the interviewers and provided with a basic background on the study and the training of the researchers prior to the interviews |
| 8. | Interviewer characteristics | What characteristics were reported about the interviewer/facilitator? e.g. *Bias, assumptions, reasons and interests in the research topic* | Affiliations of the researchers and purpose of the study was disclosed to the participants prior to the interview. |
| **Domain 2: study design** | | |  |
| Theoretical framework | | |  |
| 9. | Methodological orientation and Theory | What methodological orientation was stated to underpin the study? *e.g. grounded theory, discourse analysis, ethnography, phenomenology, content analysis* | Thematic Analysis |
| Participant selection |  |  |  |
| 10. | Sampling | How were participants selected? *e.g. purposive, convenience, consecutive, snowball* | All patients who met the eligibility criteria during the study period were included in the sample. |
| 11. | Method of approach | How were participants approached? e*.g. face-to-face, telephone, mail, email* | Participants were approached in person after admission to hospital. |
| 12. | Sample size | How many participants were in the study? | 35 |
| 13. | Non-participation | How many people refused to participate or dropped out? Reasons? | None |
| Setting |  |  |  |
| 14. | Setting of data collection | Where was the data collected? e*.g. home, clinic, workplace* | On patient wards |
| 15. | Presence of non-participants | Was anyone else present besides the participants and researchers? | Family members were occasionally present during interviews |
| 16. | Description of sample | What are the important characteristics of the sample? *e.g. demographic data, date* | Demographic data, income, employment, household composition and injury pattern |
| Data collection |  |  |  |
| 17. | Interview guide | Were questions, prompts, guides provided by the authors? Was it pilot tested? | The interview questions were adapted from the 2009/10 National Panel Survey and augmented with clinical expertise from authorship group. |
| 18. | Repeat interviews | Were repeat interviews carried out? If yes, how many? | No |
| 19. | Audio/visual recording | Did the research use audio or visual recording to collect the data? | Data was recorded using a tablet survey tool and augmented with interviewer notes |
| 20. | Field notes | Were field notes made during and/or after the interview or focus group? | Notes were made at the time of the interview and reviewed the same day to ensure all information was included. |
| 21. | Duration | What was the duration of the interviews or focus group? | Approximately 30 minutes per interview. |
| 22. | Data saturation | Was data saturation discussed? | No |
| 23. | Transcripts returned | Were transcripts returned to participants for comment and/or correction? | No |
| **Domain 3: analysis and findings**z |  |  |  |
| Data analysis |  |  |  |
| 24. | Number of data coders | How many data coders coded the data? | Two |
| 25. | Description of the coding tree | Did authors provide a description of the coding tree? | Yes |
| 26. | Derivation of themes | Were themes identified in advance or derived from the data? | Themes were derived from the data |
| 27. | Software | What software, if applicable, was used to manage the data? | Nvivo 9 Software |
| 28. | Participant checking | Did participants provide feedback on the findings? | No |
| Reporting |  |  |  |
| 29. | Quotations presented | Were participant quotations presented to illustrate the themes / findings? Was each quotation identified? e*.g. participant number* | Quotations used in the discussion were not identified to preserve confidentiality. |
| 30. | Data and findings consistent | Was there consistency between the data presented and the findings? | Yes |
| 31. | Clarity of major themes | Were major themes clearly presented in the findings? | Yes |
| 32. | Clarity of minor themes | Is there a description of diverse cases or discussion of minor themes? | Yes |
